# On Bandaging the Abdomen after Delivery

**Published:** 1852-07

**Authors:** 


*On Bandaging the Abdomen after Delivery.* By W. B. KESTEVEN, Surgeon.

[Mr. Kesteven, although sensible that the weight of opinion is against him, records his conviction that too much stress has been laid upon the importance of the bandage after delivery, and that the rationale of its usefulness has been misunderstood. In order to arrive at a correct conclusion on the subject, he examines it under the following points of view:—1st. The alleged object to be gained by the bandage. 2d. Its real effects. 3d. Its proper object, and the right period for its application. With this intent, he thus proceeds:]

1st. The objects alleged to be gained by the application of the roller directly after the completion of labor, are:—*a*, to promote the contraction of the uterus; *b*, to lessen the severity of the after-pains; *c*, to prevent hemorrhage; *d*, to prevent syncope; *e*, to protect the patient against the consequences of sudden alteration of the balance of the circulation, by which syncope, inactivity of the uterus, hemorrhage, and subsequent diseases, have been produced.

On examining, at the bedside, the validity of these several objects, it may be observed, in the first place, that all or any, of these supposed ends may be gained without the use of the bandage.

*a.* In the vast majority of cases the uterus contracts rapidly, firmly, and permanently, directly upon delivery, without the aid of bandaging. That such is the case a very short experience among the *laboring poor* will soon convince the clinical student. The poor women who are delivered by midwives, and the hundreds, ay thousands, who are yearly delivered without any aid, would, were it not so, have all the dangers of uncontracted uterus to contend with. That such is rarely the case admits of no doubt.

*b.* That measure which shall promote the contraction of the uterus can hardly be seriously recommended as a means of lessening the severity of the after-pains; the contradiction is too manifest to require further comment.

*c.* For the prevention of hemorrhage the application of a roller certainly possesses no claim. Every practitioner who has diligently applied the bandage has had to remove it, in order to apply that efficient pressure to the uterus which is most important in promoting its contractions, hemorrhage having taken place in spite of the compression that had been made by the bandage. In fact the tightly bandaging the hypogastric region with the addition of pads, compresses, basins, &c. &c., has probably frequently given rise to hemorrhage by interfering with the gradual tonic contraction of the uterus. The early application of a binder and compress is a complete obstacle to that vigilant attention to the state of the uterus after labor, which it is the wisdom as well as the duty of the medical attendant to pay for some little time after delivery. Where pressure is properly made, hemorrhage is not frequently met with. The very officious accoucheur, who loads his patient's abdomen with divers pads, and other similar contrivances, must frequently have had occasion to remove them. Without these, the earliest signs of hemorrhage may be recognized; with them, they are often concealed; without these hindrances, therefore, the occurrence may be arrested at its outset. It is not the purpose of the present communication to dwell upon the treatment of uterine hemorrhage, but the above hints may serve to show that the bandage has few claims for adoption on that score.

